# Directional deep brain stimulation of the subthalamic nucleus: A pilot study using a novel neurostimulation device

**DOI:** 10.1002/mds.26669

**Published:** 2016-05-31

**Authors:** Frank Steigerwald, Lorenz Müller, Silvia Johannes, Cordula Matthies, Jens Volkmann

**Affiliations:** ^1^ Department of NeurologyUniversity Clinic of WürzburgWürzburgGermany; ^2^ Department of NeurosurgeryUniversity Clinic of WürzburgWürzburgGermany

**Keywords:** deep brain stimulation, Parkinson's disease

## Abstract

**Introduction:**

A novel neurostimulation system allows steering current in horizontal directions by combining segmented leads and multiple independent current control. The aim of this study was to evaluate directional DBS effects on parkinsonian motor features and adverse effects of subthalamic neurostimulation.

**Methods:**

Seven PD patients implanted with the novel directional DBS system for bilateral subthalamic DBS underwent an extended monopolar review session during the first postoperative week, in which current thresholds were determined for rigidity control and stimulation‐induced adverse effects using either directional or ring‐mode settings.

**Results:**

Effect or adverse effect thresholds were modified by directional settings for each of the 14 STN leads. Magnitude of change varied markedly between leads, as did orientation of optimal horizontal current steering.

**Conclusion:**

Directional current steering through chronically implanted segmented electrodes is feasible, alters adverse effect and efficacy thresholds in a highly individual manner, and expands the therapeutic window in a monopolar review as compared to ring‐mode DBS. © 2016 The Authors. Movement Disorders published by Wiley Periodicals, Inc. on behalf of International Parkinson and Movement Disorder Society

DBS of the STN has proven to be a safe, effective treatment for patients with severe tremor, motor fluctuations, or dyskinesia in Parkinson's disease.^1^, ^2^ However, individual outcomes may vary greatly and critically depend on the brain volume being stimulated. Best motor symptom control has been associated with stimulation of the dorsolateral STN,^3^, ^4^ whereas current leaking into adjacent fiber tracts can cause adverse effects such as dysarthria, impaired fine motor control, or oculomotor disturbances.^5^

Conventional DBS systems use ring‐shaped electrodes, which generate an approximately spherical electrical field. In these systems, programming of polarity and stimulation pulse parameters allows only limited control of the shape of the volume of tissue activated.^6^ Recently, two acute intraoperative studies have proven the feasibility of horizontal current steering by using novel lead designs, such as segmented or multicontact electrodes.^7^, ^8^ Directed stimulation using these electrodes resulted in increased stimulation thresholds for side effects as compared to standard spherical stimulation.

Here, we report our first clinical experience of directional DBS with a novel, fully implantable neurostimulation system (Vercise PC; Boston Scientific, Valencia, CA), which combines eight‐contact directional leads and a pulse generator capable of multiple independent current source control (MICC). The system received a CE Mark in September 2015, and the first device was implanted at our center on 16 September 2015.

The novel directional DBS lead has four electrode levels, of which the two middle levels are split into three segments spanning approximately 120 degrees, whereas the highest and lowest level consist of ring‐shaped electrodes (Supporting Fig. 1A). MICC allows to distribute the stimulation current over any combination of electrodes of one lead in arbitrary proportions. An equal distribution of current among all three segments at one level simulates a ring‐shaped electrode, whereas maximal horizontal steering effects are obtained when current is distributed to one or two segments at one level (Supporting Fig. 1B). Our retrospective analysis of monopolar review data aimed at quantifying the effect of horizontal current steering on the therapeutic window of STN‐DBS as compared to conventional ring mode stimulation.

## Patients and Methods

Seven PD patients (2 female; age, 47–64 years; disease duration: 8–20 years; UPDRS‐III Med *off*: 42; UPDRS‐III Med *on*: 19), who had been implanted with the directional Vercise PC (Boston Scientific) for bilateral STN‐DBS between September and December 2015, underwent an extended programming session of their DBS system in the practically defined medication *off* state (>12 hours of medication withdrawal) 4 to 9 days (mean, 7 ± 2) postsurgery. The programming session was scheduled when the stun effect of electrode placement was decreasing and testable *off* period motor symptoms had returned in each patient in at least one body side.

The programming session followed the procedure of a standard monopolar review,^6^ in which for each electrode configuration current thresholds are determined for complete rigidity control (efficacy threshold) and the first adverse event (AE) limiting further current increase (AE threshold). Frequency and pulse width were constantly set to 130 Hz and 60 μs, respectively, and the implantable pulse generator case was always programmed as anode. Stimulation current was increased/decreased in steps of 0.5 mA until complete rigidity suppression (i.e., hypotonia of the upper extremity) was achieved or an AE of stimulation was reported by the patient or observed on clinical examination. Then, the current threshold was fine tuned in smaller steps of 0.1 mA.

Using this procedure, we first determined the therapeutic window (TW; current difference between efficacy and AE threshold in mA) for the two segmented levels of each lead in ring mode (equally distributing current among the three segments of a level) and labeled the level with the larger TW as “most effective ring level.” Then, different stimulation directions at each level were tested by either restricting cathodal current to each of the three segments (first patient) or additionally evaluating equal current distributions between two adjacent segments (subsequent 6 patients). This programming results in up to six directed electrical fields with a field vector rotated by either 120 or 60 degrees (Supporting Figs. 1B and 2). The sequence of levels or horizontal directions tested was left to the programming physician's discretion, who was unaware of the anatomical position and orientation of the lead within STN. Threshold amplitudes were compiled in a datasheet and later used for constructing individual polar plots (see Supporting Fig. 2) and descriptive statistics (paired two‐sample *t* test).

## Results

We could determine efficacy thresholds for only 11 of 14 STNs, because a persistent microlesioning effect prevented reliable rigidity assessment in the others, whereas AE thresholds were determined for all 14 STNs. The AEs determining the upper limit of the TW were contractions of facial or hand muscles in 11 of 14 STNs, dysarthria in 6 of 14, and persisting dysesthesia in 1 of 14.

In total, we assessed 154 directional and 28 ring‐mode settings and determined TW for 111 and 24 settings, respectively. For each directional setting, the proportional change of TW from corresponding ring‐mode stimulation was calculated, with negative values indicating a reduction and positive values indicating an increase of the TW. The proportional change was highly variable between leads and directional settings and ranged between –100% and 440% (see Table 1). Interestingly, this change in TW was not only determined by a variable AE threshold, but also by variations in the effect threshold (see Supporting Fig. 3).

**Table 1:** Best and worst effect on TW

		Most Effective Level					Less Effective Level				
ID	Side	At level	Best ΔTW (%)	Best direction	Worst ΔTW (%)	Worst direction	At level	Best ΔTW (%)	Best direction	Worst ΔTW (%)	Worst direction
01	Left	5‐6‐7	35	post‐med	–42	post‐lat	2‐3‐4	57	post‐med	–14	post‐lat
01	Right	13‐14‐15	9	ant	–9	post‐lat	10‐11‐12	9	ant	–22	post‐med
02	Left	5‐6‐7	28	post‐med	–48	post‐lat	2‐3‐4	440	post‐med	–100	post
02	Right	10‐11‐12	10	ant	–50	post‐med	13‐14‐15	100	ant	–20	post
03	Right	10‐11‐12	–6	ant‐med	–65	post‐lat	13‐14‐15	–3	ant	–100	post
04	Right	10‐11‐12	27	post‐med	–44	ant‐lat	13‐14‐15	77	post‐med	0	ant‐lat
05	Left	2‐3‐4	88	med	0	post‐lat	5‐6‐7	171	ant‐med	29	ant
05	Right	13‐14‐15	–5	post	–52	ant‐med	10‐11‐12	135	ant‐lat	41	ant
06	Right	13‐14‐15	–9	lat	–75	post‐med	10‐11‐12	47	ant	–89	post‐med
07	Left	2‐3‐4	–8	ant‐med	–72	post‐lat	5‐6‐7	135	post	47	lat
07	Right	13‐14‐15	9	post	–18	post‐lat	10‐11‐12	50	ant‐lat	0	post

Changes in therapeutic window (ΔTW) in best and worst direction and respective orientation of electrical field vector are given for all STN in which effect and AE threshold could be determined.

post‐med, posteromedial; ant, anterior; ant‐med, anteromedial; med, medial; post, posterior; lat, lateral; post‐lat, posterolateral; ant‐lat, anterolateral.

By fusing the postoperative cranial CT with the preoperative MRI (Leksell Surgiplan; Elekta Instrument AB, Stockholm, Sweden) and identifying the directional lead marker, we determined the orientation of each lead within stereotactic space and assigned each directional setting to an orientation in relation to the anterior commissure/posterior commissure (AC‐PC) line (e.g., anterior, anterolateral, etc.). This allowed us to identify the anatomical direction of the current vector providing the highest positive change in TW (best orientation) and the smallest change (worst orientation) for each lead and level. As expected from variable lead locations within a variably shaped and oriented STN, there was no uniform best direction, and best versus worst orientation were often, but not always strictly opposite (see Table 1). Most often, the “optimal” field vector was oriented in an “anterior” or “posteromedial” direction.

**Figure 1:**
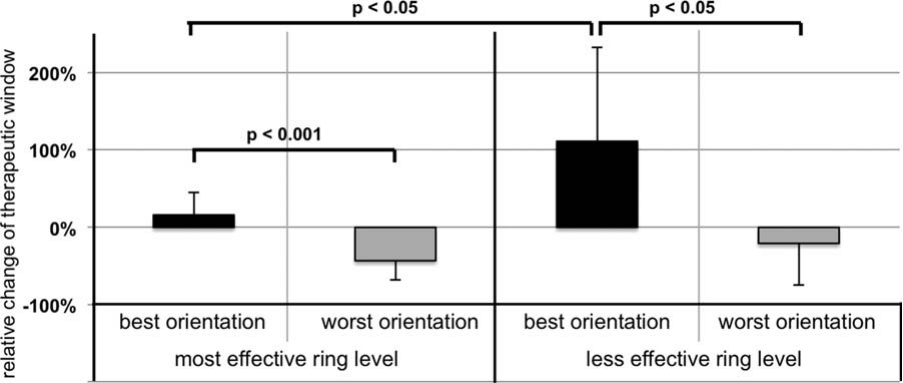
Bar graph depicting the relative change of the therapeutic window (%) when steering current in the best vs. worst orientation during the monopolar review. Please note, the change in therapeutic window for best directional current steering was proportionally larger at the less effective ring level. On both levels therapeutic windows assessed for the best orientation of current steering differed significantly from those obtained when stimulating into the worst orientation highlighting a potential clinical usefulness of directional DBS.

After grouping the results of directional stimulation by ring level, it became apparent that larger TW effects of optimal current steering could be observed at the less effective (111 ± 122%; median, 77; range, –3 to 440) as compared to the most effective level (16 ± 22%; median, 9; range, –9 to 88%; see Fig. 1). The notion of a larger TW with directional DBS is also supported by a secondary analysis comparing the amplitude range between efficacy and AE threshold of optimal current steering at the most effective level compared to ring mode (4.0 ± 1.5 vs. 3.6 ± 1.4 mA; *P* = 0.06).

Interestingly, the change in TW was driven only by an increase in AE threshold at the most effective level, whereas at the less effective level, both efficacy and AE thresholds, changed significantly in favor of the optimal direction (1.25 ± 1.23 vs. 1.77 ± 0.95 mA; *P* = 0.05; 5.39 vs. 4.27 ± 1.46 mA; *P* < 0.005)

At the end of the monopolar review, the optimal directional settings were programmed in all patients and gradually adjusted according to clinical needs during the subsequent days of hospitalization. After a follow‐up of 3 to 6 months (median, 4), all patients have remained programmed in directional mode without need of rescue programming into ring mode to improve stimulation efficacy (see Supporting Table 1). None of the patients are complaining about stimulation induced adverse effects so far.

## Discussion

Our results demonstrate, for the first time, the feasibility of horizontal current steering using a fully implanted neurostimulation device with directional leads and MICC technology. Despite the limitations of an acute monopolar review in the early postoperative period, they also provide first evidence of a beneficial impact of directional DBS on the TW. In theory, a larger TW would offer more programming flexibility for optimizing the efficacy of DBS and reduce the likelihood of inadvertently exceeding the adverse effect threshold, when the stimulation amplitude is gradually adjusted during the subsequent stabilization period.^6^ Hence, directional DBS should result in more consistent good outcomes and lower AE rates across groups of patients, whereas it is unlikely to provide more benefit than an optimally implanted ring‐mode DBS in an individual.

Not unexpectedly, we found a larger effect of directional DBS at the less beneficial lead level. This indicates that the individual clinical benefit of directional DBS is best observed for suboptimal electrode positions resulting in a narrow TW, for example, if the electrode is placed too laterally within the STN close to the internal capsule. Hence, directional DBS may be able to compensate within certain limits for small deviations of the lead from the optimal functional target, which are a main source of outcome variability in STN‐DBS even in experienced surgical centers.^4^ However, as a note of caution, the availability of a directional DBS system must never be an excuse for lowering the surgical standard and precision of surgical lead placement. In fact, the implantation of the Vercise directional lead is surgically more challenging, because the active site has a reduced span, compared to the standard eight‐contact ring lead, and the split contacts need to be exactly aligned in depth with the dorsolateral motor region of the STN. Moreover, lead rotation is introduced as an additional degree of freedom during the implantation and needs to be controlled for by exact alignment of the rotational lead marker with patient centric landmarks (e.g., AC‐PC line).

Other limitations of our study include the unblinded and subjective clinical rating of rigidity and adverse effect thresholds, lack of long‐term clinical follow‐up, early postoperative time period with a partially persistent stun effect, and small number of subjects. Importantly, we report feasibility data obtained during an acute stimulation challenge, but no efficacy data on the use of chronic directional DBS compared to standard ring DBS. Nevertheless, our findings may provide valuable input into the planning of appropriate clinical trials, which are now needed to establish the theoretical advantages of directional DBS in clinical practice and on a group level, but should also take into account possible disadvantages of the expanded parameter space, such as increased programming burden.

## Author Roles

(1) Research Project: A. Conception, B. Organization, C. Execution; (2) Statistical Analysis: A. Design, B. Execution, C. Review and Critique; (3) Manuscript Preparation: A. Writing of the First Draft, B. Review and Critique.

F.S.: 1A, 1B, 1C, 2A, 2B, 2C, 3A, 3B

L.M.: 1B, 3B

S.J.: 1C, 3B

C.M.: 1C, 3B

J.V.: 1A, 1C, 2A, 2B, 2C, 3A, 3B

## Financial Disclosures

F.S. has been a consultant for Boston Scientific and has received travel and speaker honoraria from Medtronic and St. Jude Medical. C.M. has been a consultant for Boston Scientific. J.V. has received grants and personal fees from Medtronic and Boston Scientific and personal fees from St. Jude, UCB, Merz, Allergan, TEVA, Novartis, and AbbVie.

## Supporting information

Additional Supporting Information may be found in the online version of this article at the publisher's web‐site.

Supplementary Information
