# Supplementary material for: Directional deep brain stimulation of the subthalamic nucleus: A pilot study using a novel neurostimulation device
Source: Mov Disord. 2016 May 31;31(8):1240–3. doi: 10.1002/mds.26669 (PMC5089579; doi:10.1002/mds.26669)
Supplement: Supplementary file 1 — Supplementary Information [file MDS-31-1240-s001.docx]

**Supplement**

**
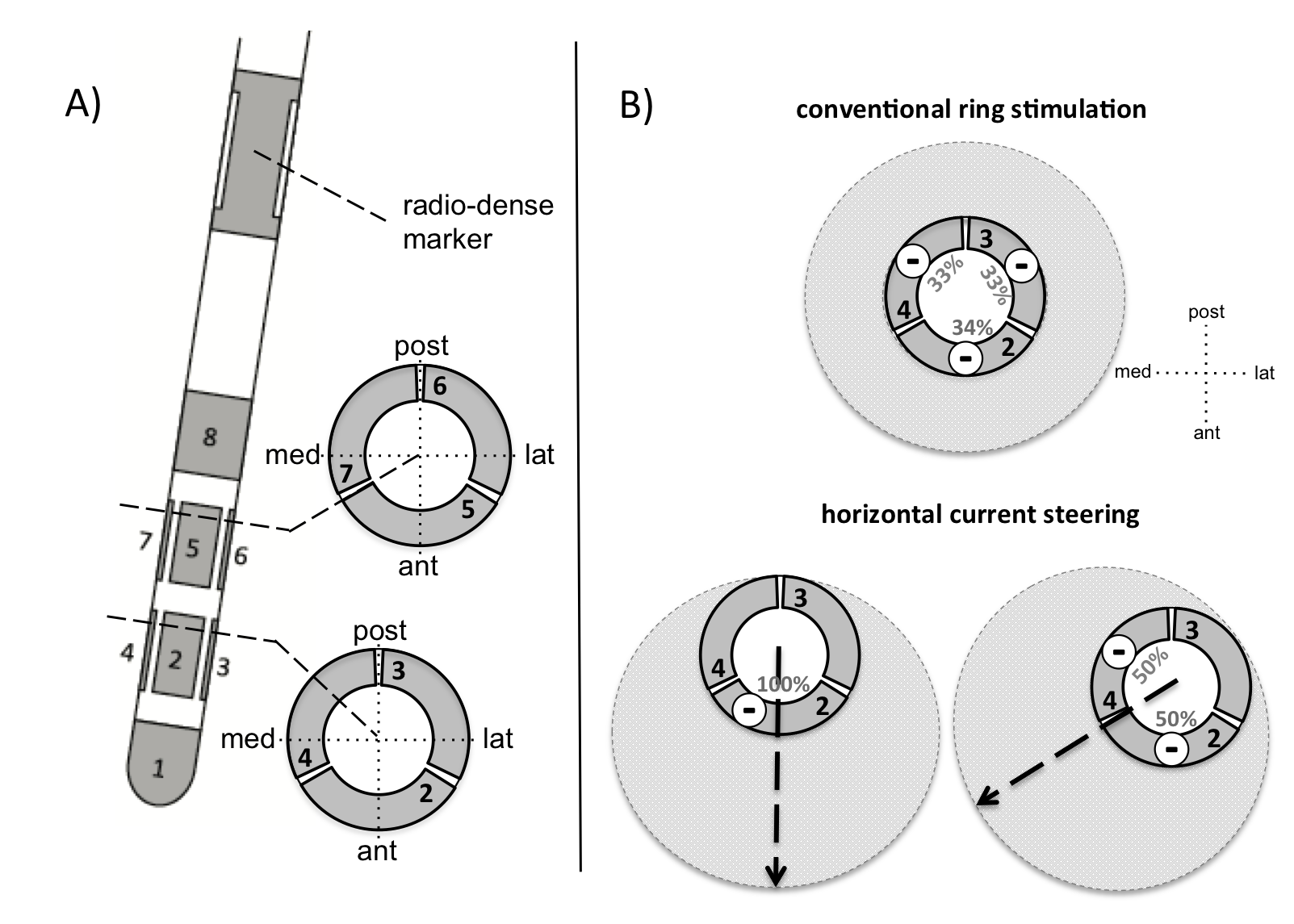
**

**Figure 1) Scheme of the directional electrodes with segmented contacts**

1. Example of L STN electrode with an active tip contact (1; R STN 9), 2 levels with 3 segmented 120° contacts (2-4, 5-7; R STN 10-12, 13-15) and 1 conventional ring electrode above (8; R STN 16). A radiodense marker aligned to contacts 2 and 5 helps to control intraoperative anterior orientation by fluoroscopy.
2. Estimate model of the electric field in conventional ring mode distributing the current outflow equally to all three contacts of one level (above) or in directional mode (above). By restricting the current to just one contact (below L) or in 50:50% distribution on two neighbouring contacts the vector of the electrical field can be moved in 60° steps

**
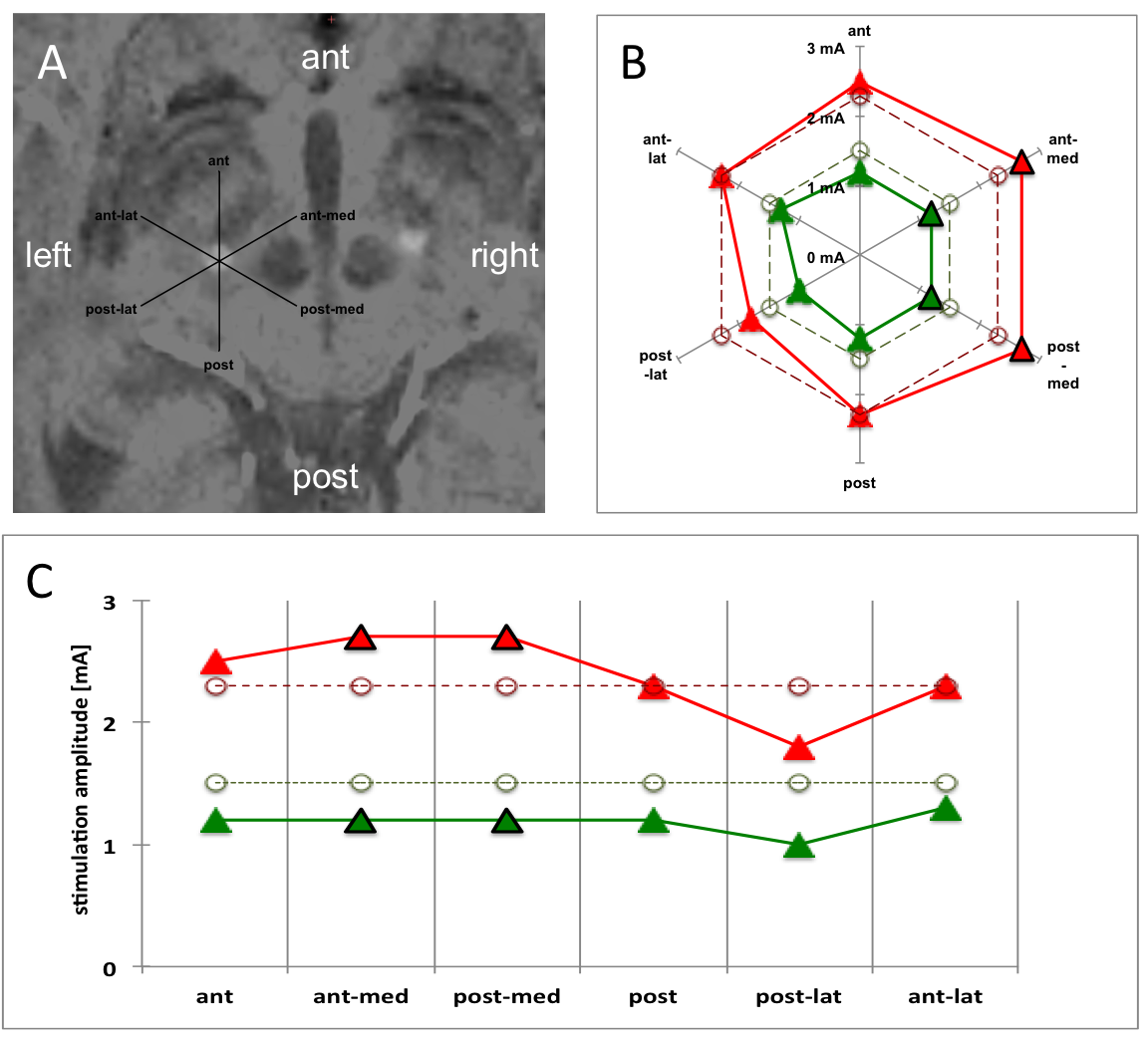
**

**Fig. 2) Orientation of directional lead relative to STN and threshold plots**

1. Fusion of postoperative CCT and preoperative SWI-MRI shows localization of L STN-electrode in the posterior part of the STN. Directions tested in directional mode depicted in the image
2. Radar plot of effect and adverse effect threshold in conventional ring mode (dashed lines and open markers in dark green = effect and dark red = adverse effect) and superimposed thresholds in directional mode (continuous lines and solid markers in green = effect and red = adverse effects) tested in 60° steps. Directions with largest therapeutic windows are marked by black marker outlines; in this example a medial direction.

C) For better visualisation and comparison between different STN radar plots were converted to line plot (same colour coding as in B)

**
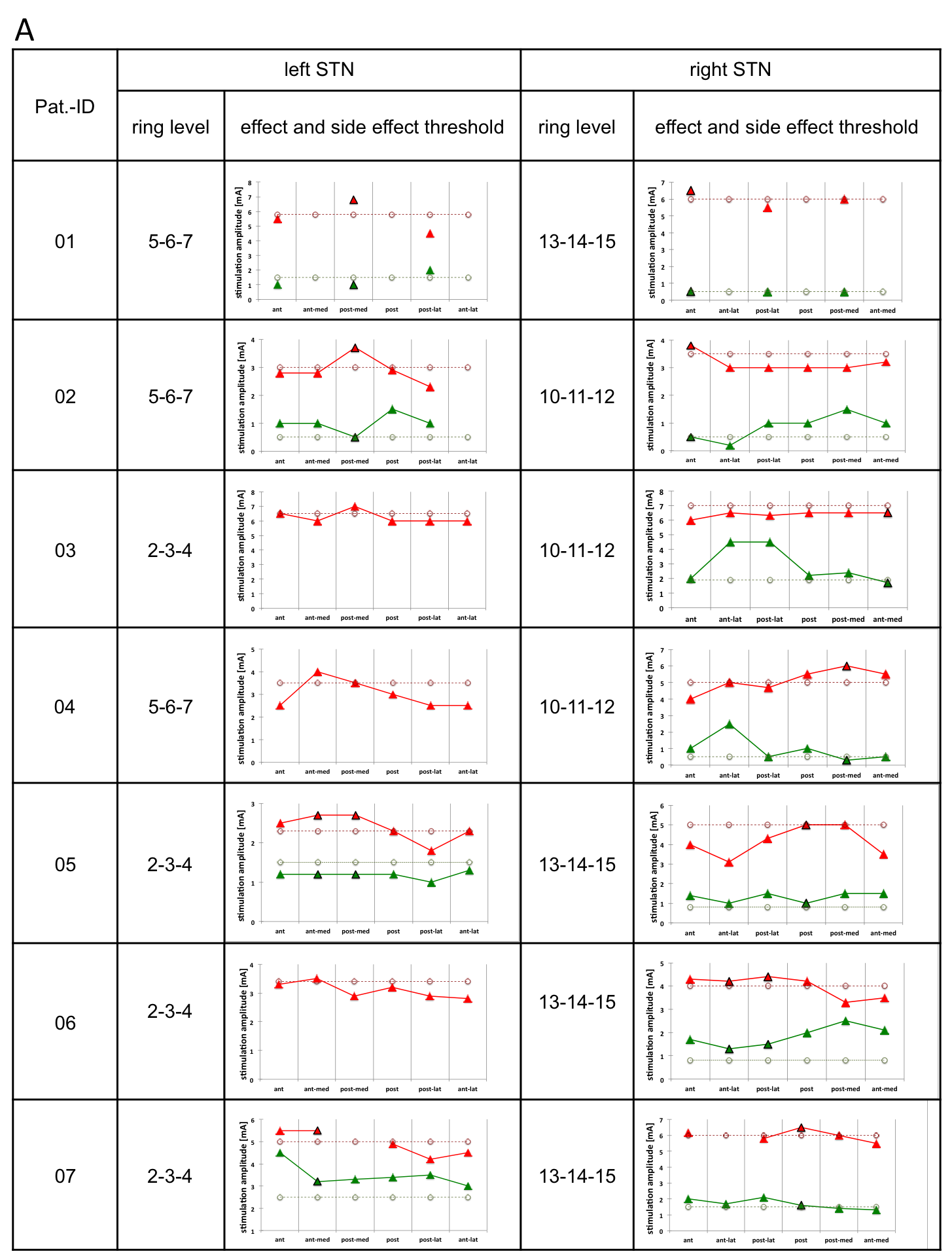
**

**
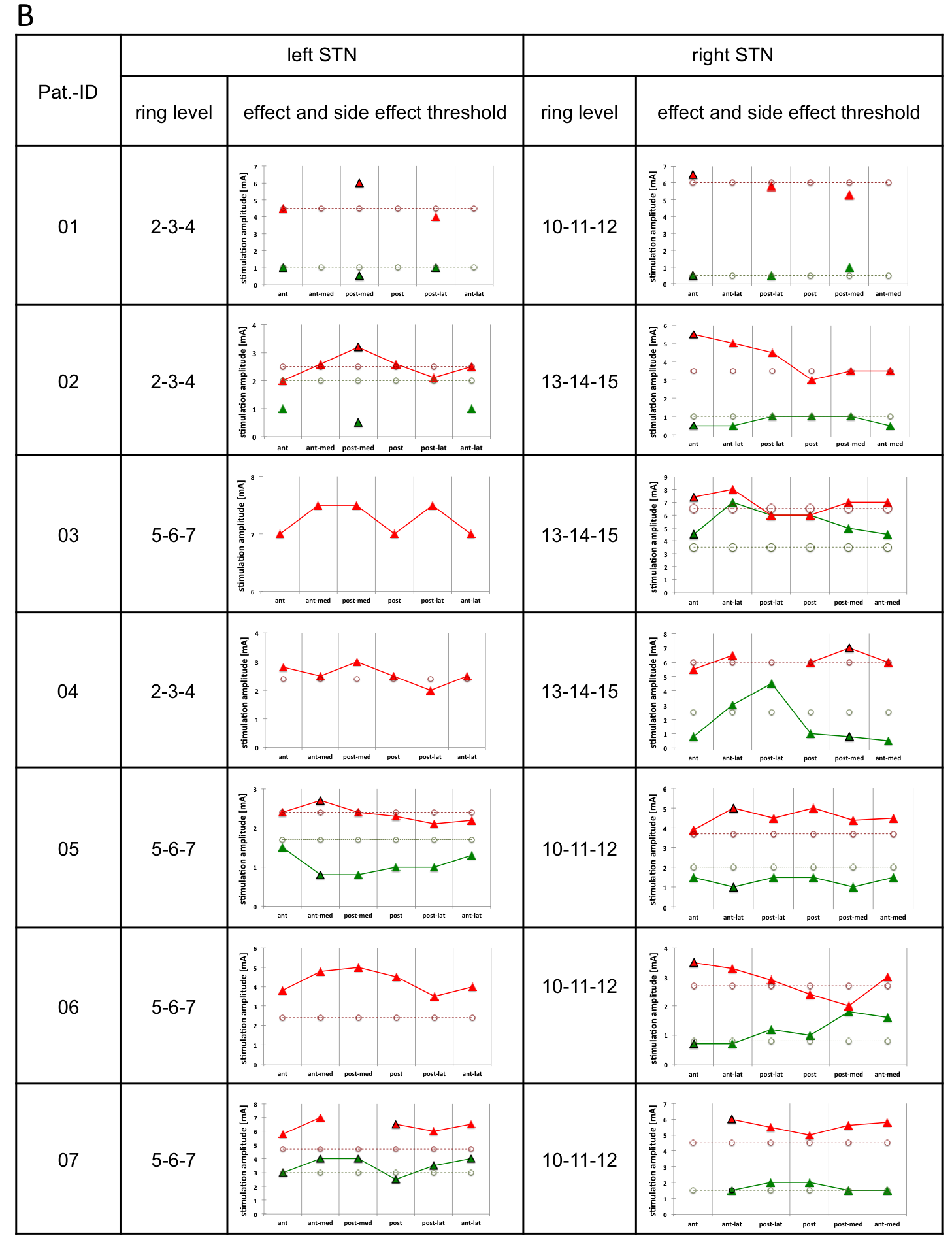
**

**Fig. 3) Line plots of effect and adverse effect threshold of all STN**

Effect and adverse effect threshold plots of all STN at the most (A) and less effective (B) ring level. Ring mode threshold are drawn with broken lines and open markers, directional mode threshold with solid lines and markers. Effect thresholds in green, adverse effect thresholds in red. Directions with largest therapeutic windows marked by black marker outlines.

| **Pat ID** | **UPDRS III pre-OP** | | **last follow up** | | **side** | **DBS type** | **Amp. [mA]** | **Pulse width [µs]** | **Frequency [Hz]** |
| --- | --- | --- | --- | --- | --- | --- | --- | --- | --- |
|  | **OFF** | **ON** | **day** | **UPDRS III** |  |  |  |  |  |
| **01** | 44 | 15 | 146 | 19 | L | horizontal & vertical steering | 2.5 | 60 | 154 |
|  |  |  |  |  | R | horizontal & vertical steering | 4.0 | 60 | 154 |
| **02** | 39 | 11 | 128 | 14 | L | horizontal & vertical steering | 4.5 | 60 | 79 |
|  |  |  |  |  | R | horizontal & vertical steering | 4.3 | 30 | 79 |
| **03** | 42 | 21 | 132 | 12 | L | horizontal steering | 3.0 | 60 | 179 |
|  |  |  |  |  | R | horizontal & vertical steering | 3.5 | 60 | 179 |
| **04** | 40 | 19 | 85 | 18 | L | horizontal steering | 1.8 | 60 | 130 |
|  |  |  |  |  | R | horizontal steering | 2.5 | 60 | 130 |
| **05** | 85 | 42 | 98 | 34 | L | horizontal steering | 1.9 | 60 | 130 |
|  |  |  |  |  | R | horizontal steering | 1.7 | 60 | 130 |
| **06** | 51 | 11 | 80 | 17 | L | horizontal steering | 3.5 | 60 | 130 |
|  |  |  |  |  | R | horizontal steering | 2.0 | 60 | 130 |
| **07** | 45 | 27 | 80 | 23 | L | horizontal & vertical steering | 3.5 | 60 | 149 |
|  |  |  |  |  | R | horizontal steering | 2.0 | 40 | 179 |

**Tab. 1) Follow up data**

Comparison of preoperative MDS-UPDRS-III scores in medication OFF and best medication ON with ON during last follow up shows good efficacy of DBS in all patients. All patients remained on horizontal steering, in 6 of 14 hemisphere directional (= horizontal) current steering was combined with some degree of vertical current steering to the adjacent level (L = left hemisphere; R = right hemisphere).
